# Lipid-Lowering Effects of *Inonotus obliquus* Polysaccharide In Vivo and In Vitro

**DOI:** 10.3390/foods10123085

**Published:** 2021-12-12

**Authors:** Mo Yang, Dong Hu, Zhengying Cui, Hongxuan Li, Chaoxin Man, Yujun Jiang

**Affiliations:** 1 Key Laboratory of Dairy Science, Department of Food Science, Ministry of Education, Northeast Agricultural University, Harbin 150030, China; yang1994mo@163.com (M.Y.); 18346731635@163.com (Z.C.); hongxuan_li@yeah.net (H.L.); cxman@neau.edu.cn (C.M.); 2 Institute of Genetics and Physiology, Hebei Academy of Agriculture and Forestry Sciences, Shijiazhuang 050051, China; donghu1983@163.com

**Keywords:** *Inonotus obliquus* polysaccharide, HepG2 cells, high-fat diet, lipid lowering

## Abstract

Excessive lipid intake will cause hyperlipidemia, fatty liver metabolism disease, and endanger people’s health. Edible fungus polysaccharide is a natural active substance for lipid lowering. In this study, the HepG2 cell model induced by oleic acid and mice model induced by a high-fat diet was established. The lipid-lowering effects of *Inonotus obliquus* polysaccharide (IOP) was investigated in vivo and in vitro. Glucose (251.33 mg/g), rhamnose (11.53 mg/g), ribose (5.10 mg/g), glucuronic acid (6.30 mg/g), and galacturonic acid (2.95 mg/g) are present in IOP, at a ratio of 85.2:3.91:1.73:2.14:1. The molecular weight of IOP is 42.28 kDa. Treatment with 60 mg/L of IOP showed a significant lipid-lowering effect in HepG2 cells compared with the oleic acid-treated group. In the oil red O-stained images, the red fat droplets in the IOP-treated groups were significantly reduced. TC and TG levels of IOP-treated groups decreased. IOP can alleviate the lipid deposition in the mice liver due to high-fat diet, and significantly reduce their serum TC, TG, and LDL-C contents. IOP could activate AMPK but decrease the SREBP-1C, FAS, and ACC protein expression related to adipose synthesis in mice. IOP has a certain potential for lipid-lowering effects both in vivo and in vitro.

## 1. Introduction

With the improvement of people’s living standards, the intake of high-fat diets is increasing. Excessive lipid intake can cause many metabolic diseases. Excessive release of saturated-fatty acids, refined carbohydrates, fructose, and high caloric intake in diets promote obesity and non-alcoholic fatty liver disease [1]. Excessive free fatty acids, excessive sugar intake, and oxidative damage are the main reasons of metabolic diseases [2]. Moreover, lipid accumulation is closely related to metabolic syndromes such as hypertension, hyperlipidemia, and non-alcoholic fatty liver disease, so it has attracted widespread attention [3]. Lipid metabolism in normal liver cells is in a balanced state, but when lipids accumulate too much, liver cells will experience steatosis. Currently, reducing food intake, losing weight, and removing excessive fat surgically may alleviate lipid accumulation. However, surgery is harmful to the human body. In this context, a substance that has the potential to reduce liver lipids is urgently needed. The development of functional polysaccharides may be one of the safe treatments for anti-dyslipidemia [4].

Polysaccharides are mainly extracted from plants, animals, or microorganisms. Polysaccharides have been extensively studied because of their functional properties such as anti-inflammatory and anti-tumor [5,6]. Previous studies have shown that mushroom polysaccharides have safe natural hypolipidemic effects [7,8]. Polysaccharides extracted from *Cordyceps militaris* have potential hypolipidemic activity in vitro [9]. In addition, *Pleurotus eryngii* polysaccharides could reduce TC (Total cholesterol) and TG (Total triglycerides) contents in mice serum [10]. *Inonotus obliquus*, also known as chaga, mainly grows in cold areas, for example in northeast China, northern Europe, and Russia [11]. *I. obliquus* are born under the bark of silver birch, elm, and alder. The extracts of *I. obliquus* have favorable effects, as it can promote lipid metabolism and modulate cardiac function [12]. Previous studies have shown that *I. obliquus* is rich in oligosaccharides and polysaccharides [13]. *I. obliquus* polysaccharide is the main substance with biological activity [14]. Studies have indicated that *I. obliquus* polysaccharide (IOP) is a new natural anti-hyperglycemic and anti-fatigue substance [15,16]. According to previous studies, IOP may have lipid-lowering function; however, information about lipid accumulation inhibition of IOP is currently limited.

Adenosine monophosphate activated protein kinase (AMPK) is an energy sensor discovered in recent years. It is considered to be a key enzyme protein that regulates cell metabolism, regulates fat content, and affects lipid metabolism balance [17]. Sterol regulatory element binding protein (SREBP-1C) is an important transcription factor that regulates lipid de novo synthesis, and its downstream key target genes also include acetyl Co A carboxylase (ACC) and fatty acid synthases (FAS) [18,19]. Therefore, based on the key enzymes of AMPK/SREBP-1C, ACC, and FAS that regulate the important signaling pathways of lipid metabolism, we need to explore the regulating and controlling lipid metabolism mechanism of IOP.

In this study, the structure of IOP was characterized. The effects of IOP in HepG2 cells under conditions of excessive fatty acids induced by oleic acid (OA) were evaluated. The effects of IOP on the blood lipid levels and lipid metabolism-related genes and proteins in mice induced by high-fat diet were investigated. This study provides a research foundation for clarifying the material basis and related mechanisms of IOP for reducing blood lipids.

## 2. Materials and Methods

### 2.1. Materials

*I. obliquus* was purchased from Daxinganling (Heilongjiang, China). HepG2 cells were preserved in the cell laboratory of Northeast Agricultural University. The 6-well plates were purchased from Falcon (Beijing, China). DMEM, FBS, and PBS were purchased from Thermo Fisher Scientific Co., Ltd. (Shanghai, China). MTT and DMSO were purchased from BioFroxx (Yucheng Biotechnology Co., Ltd. Nanjing, China). Total RNA Extraction kit was purchased from Bioer Technology (Beijing, China). Reverse transcription kit was purchased from Takara Bio (Shiga, Japan). The other kits were purchased from Nanjing Jiancheng Bioengineering Institute (Jiangsu, China). Other reagents were purchased from Solebold Technology Co., Ltd. (Beijing, China).

### 2.2. IOP Preparation

The polysaccharide was extracted as Xu’s method with slight modifications [20]. *I. obliquus* powder was extracted using hot distilled water. Ethanol (95%) was added to the supernatant in a volume ratio of 1:3 (The final alcohol concentration was adjusted to 80%) for 12 h. The precipitation was deproteinized three times using trichloroacetic acid (TCA) method with 5% TCA solution (precipitation: TCA solution = 1:1.5). The supernatant was concentrated and freeze dried to obtain IOP (the response surface optimization of IOP preparation conditions is shown in the Supplementary File.)

### 2.3. Characterization of IOP

#### 2.3.1. UV-Vis Analysis of IOP

UV-Vis Spectrometer (T6, Purkinje General Instrument Co. Ltd., Beijing, China) was used to detect the spectrum of IOP solution (0.1 mg/mL) in the range of 200–400 nm.

#### 2.3.2. Molecular Weight of IOP

The molecular weight of IOP was measured by GPC ELEOS System (Wyatt Technologies Inc., Goleta, CA, USA) with Shodex OHpak series SB-806 HQ column following previously described methods with some modifications [21]. Ultrapure water with 0.02% NaN_3_ was used as mobile phase, injection volume was set as 500 μL, flowrate was set as 1.0 mL/min, and the column temperature was set as 40 °C.

#### 2.3.3. Monosaccharide Composition of IOP

PMP pre-column derivatization method [22] with some modifications was used to determine the monosaccharide composition of IOP. At 120 °C, IOP (10 mg) was hydrolyzed with 1 mL of TFA (4 mol/L) for 2 h, and the above hydrolysate was blown dry with nitrogen. Then, 1 mL of 0.5 mol/L PMP-methanol solution was added to the dried sample with 0.5 mL of 0.3 mol/L NaOH solution in 70 °C for 60 min. The sample cooled down naturally, HCl solution (0.5 mL of 0.3 mol/L) and chloroform (0.5 mL) were added into the samples, and the lower layer was discarded 20 min later. Exaction process were repeated three times, and the water layer was passed through the membrane. The components were separated using a SHISEIDO C18 column (column temperature was 25 °C). The sample injection volume was 10 μL. KH_2_PO_4_ and acetonitrile (ratio: 82:18) were used as mobile phase with flow rate of 1.0 mL/min. The data were measured at 245 nm wavelength.

#### 2.3.4. FTIR Spectrum Analysis

IOP (1 mg) was ground with KBr (100 mg) and tableted into pellets. The data of the FTIR spectrometer (Bruker, Frankfurt, Germany) in the wavenumber range of 4000–400 cm^−1^ was recorded.

#### 2.3.5. SEM Analysis

IOP was observed IOP was placed on the copper post and sputtered with gold. At 15 V, the morphologies of IOP under 500×, 1000×, and 1500× magnification were observed using SEM (TM-3000 scanning electron microscope, Hitachi, Ltd., Tokyo, Japan).

### 2.4. Cell Culture

HepG2 cells (1 × 10^5^ cells) were resuscitated in a cell culture flask and incubated in a DMEM-high glucose (25 mmol/L, 5 mL) medium added 20% FBS, 75 μg/mL 1% streptomycin, and 100 U/mL penicillin. HepG2 cells were cultured at 95% humidity, 37 °C, and 5% CO_2_ conditions for 48h. The cell culture medium was changed into fresh DMEM-high glucose (25 mmol/L, 5 mL) medium with 10% FBS, penicillin and streptomycin, after the six-well plate washed with PBS twice. HepG2 cells were incubated at the same conditions as above and subcultured when the cells reached 80% confluence.

### 2.5. Cell Viability

According to Xiao’s method, cell viability was tested by MTT assay [23]. The high-glucose DMEM containing OA (0, 0.1, 0.2, 0.3, 0.4, and 0.5 mM) and IOP (20, 40, 60, 80, and 100 mg/L) were added to a 6-well plate and HepG2 cells were incubated for 24 h (37 °C, 5% CO_2_). Then, the 6-well plate were washed twice with PBS. Thereafter, MTT solution (5 mg/mL, 20 μL) and HepG2 cells were incubated together. After 4 h, MTT solution was discarded, 150 μL of DMSO was added and reacted for 15 min. The OD value was measured at 570 nm. The cell viability was calculated by the percentage of absorbance relative to control.

### 2.6. Oil Red O Staining for OA-Induced HepG2 Cells

Cell concentration was adjusted to 1 × 10^5^ cells/mL, 2 mL cell culture was inoculated per well into a 6-well plate and cultivated for 24 h to adhere to the wall. Intracellular lipid was determined by Oil red O staining according to Zhu’s method with slight modifications [24]. The cell status was observed under an inverted microscope.

### 2.7. TC, TG, HDL-C, and LDL-C Contents of OA-Induced HepG2 Cells

After the cell reaches a polarized state, HepG2 cells were divided into five groups: NC group, OA group (0.2 mM OA), LIOP (0.2 mM OA + 20 mg/L), MIOP (0.2 mM OA + 40 mg/L), and HIOP (0.2 mM OA + 60 mg/L). The TC, TG, HDL-C, and LDL-C contents of each group were determined according to the kits.

### 2.8. Animals and Treatment

After forty C57BL/6J mice (20 ± 2 g, 4-week-old) were adapted to feeding for 1 week, they were randomly divided into five groups with eight mice in each group, namely the normal control group (NC), high-fat diet model group (HFD), low-dose *Inonotus obliquus* polysaccharide group (LIOP), medium-dose group (MIOP), and high-dose group (HIOP). The NC group was fed an ordinary feed, and other groups were fed a high-fat diet. During the 10-week experiment, the mice were free to eat and drink water. The specific gavage methods are shown in Table 1. During the experiment, the body weight of mice was weighed once a week and recorded. After gavage for 10 weeks, the mice were fasted with water for 12 h, blood was taken from the eyeballs, and the cervical vertebrae were dislocated to death. Then, the liver was put in liquid nitrogen and stored at −80 °C for further use. The animal experiment was approved by the Laboratory Animal Welfare and Ethics Committee of the Northeast Agricultural University, and conducted according to the Guide for the Care and Use of Laboratory Animals (China).

### 2.9. Determination of Biochemical Indicators of Mice Serum

The collected mice blood samples were centrifuged at 3000 r/min and 4 °C for 10 min, and the serum was separated. The concentrations of TC, TG, LDL-C, and HDL-C were determined according to the kit instructions.

### 2.10. Liver H&E Staining

Liver tissue (size of 0.5 × 0.5 × 0.5 cm^3^) of each group was fixed with 4% paraformaldehyde solution, embedded in paraffin, and HE stained. The morphological changes of liver tissue were observed under an upright microscope [25].

### 2.11. Determination of mRNA Expression by RT-PCR

The cycling conditions and calculation methods of RT-PCR refer to Sun’s method [26]. Total RNA from HepG2 cells was extracted. Reverse transcription of RNA into cDNA was carried out according to the kit operating instructions. The primers used in the experiment are listed in Table 2.

### 2.12. Determination of Protein Expression by Western Blotting

RIPA Lysis Buffer (1 mL) was added to 0.1 g of liver tissue. The homogenate was incubated on ice for 30 min, and then centrifuged at 13,000 rpm at 4 °C for 5 min. The supernatant was the total protein solution. The protein concentration was determined by BCA protein kit. The protein was separated by SDS-PAGE and transferred to the PVDF membrane. The transferred membrane was added to the blocking solution and blocked at room temperature for 1 h. After removing the blocking solution, the membrane and primary antibody were kept overnight at 4 °C. The membrane was washed in TBST for 5 min, which was repeated three times. Then, the diluted secondary antibody was incubated at room temperature for 30 min, washed with TBST on a shaker at room temperature for 5 min, which was repeated four times. Protein bands were visualized by ECL using Image J software.

### 2.13. Statistical Analysis

For cell experiments, six replicate wells were set up. For mice experiments, each group had eight mice. The experimental data were calculated by SPSS software 22.0 (IBM Corporation, Armonk, NY, USA) for significance analysis. *p* < 0.05 means significant, *p* < 0.01 means extremely significant. The data were expressed as mean ± standard deviation (SD).

## 3. Results

### 3.1. Preparation of IOP

Hot water extraction is an efficient preparation method for IOP. The optimal extraction conditions were as follows: time of 3.06 h, temperature of 80.16 °C, and water material ratio of 30.81:1. Under the conditions, the yield of IOP was 5.37% ± 0.69% (response surface optimization of IOP preparation conditions was shown in the Supplementary File.)

### 3.2. IOP Characterization

**Figure 1:**
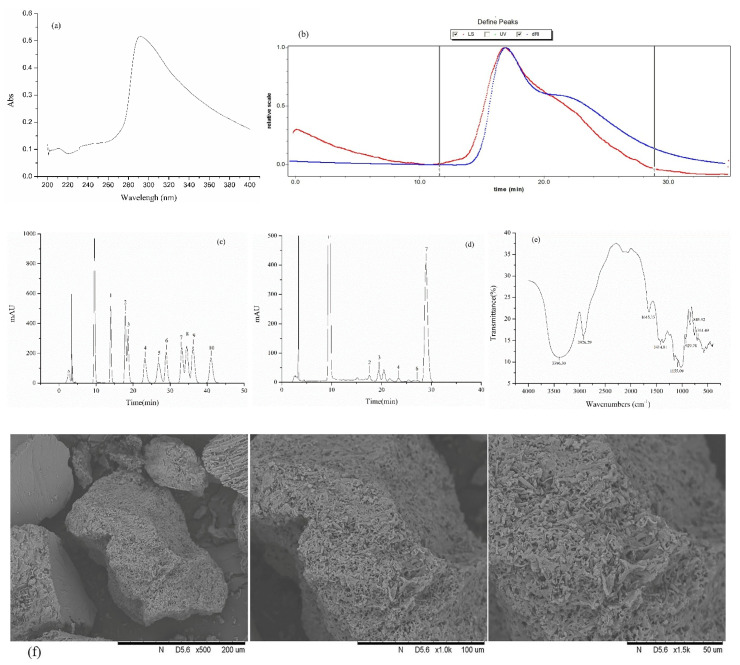
Preliminary characterization of IOP. (**a**) UV–Vis analysis of IOP. (**b**) Molecular weight of IOP. (**c**) The standard monosaccharide. (**d**) The monosaccharide composition of IOP. 1. D—mannose, 2. D—ribose, 3. L—rhamnose, 4. D—glucuronic acid, 6. D—galacturonic acid, 7. D—glucose, 8. D—galactose, 10. D—xylose, 11. L—Arabinose, 12. L—fucose. (**e**) FTIR analysis of IOP. (**f**) The SEM images of IOP observed at magnifications of 500×, 1000×, and 1500×.

No peaks were detected at 260 and 280 nm, indicating that there were no proteins or nucleic acids presented in IOP (Figure 1a). The molecular weight of IOP was determined by GPC. The estimated Mw and Mn values of IOP were 42.28 and 38.44 kDa, respectively (Figure 1b). The Mw/Mn value of IOP was 1.10, representing the narrow molecular weight distribution. The monosaccharide composition of IOP is shown in Figure 1d. IOP is a heteropolysaccharide. Glucose (251.33 mg/g), rhamnose (11.53 mg/g), ribose (5.10 mg/g), glucuronic acid (6.30 mg/g), and galacturonic acid (2.95 mg/g) were present in IOP and the ratio was 85.2:3.91:1.73:2.14:1. The FTIR spectrum of IOP in the range 4000–400 cm^−1^ was shown in Figure 1e. The absorption peak at 3396.3 and 2926.29 cm^−1^ corresponded to the stretching vibration of O–H and C–H, respectively. The absorption peaks at 1645.13 and 1414.81 cm^−1^ can be attributed to N–H and C–O, respectively. The absorption peak at 1155.09 cm^−1^ represented the pyranose-type glucose conformation. In addition, the absorption peaks at 929.78 and 761.49 cm^−1^ represented the D–glucopyranose ring antisymmetrical vibration and symmetrical vibration. The peak of 845.52 cm^−1^ represented the β–D–glucosidic linkages. IOP was observed under 500×, 1000×, and 1500× magnification by SEM. Figure 1f shows that the shape of IOP was irregular, and its surface was rough and porous. IOP is an acidic polysaccharide, which may have good biological activity.

### 3.3. Effect of IOP on the Viability of the OA-Induced HepG2 Cell Model

**Figure 2:**
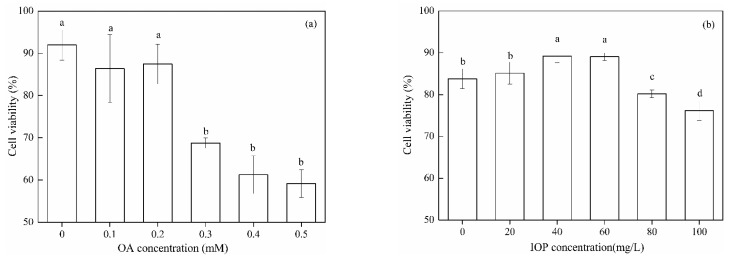
Cell viability of HepG2 cells. (**a**) Effect of OA on the cell viability of HepG2 cells. (**b**) Effect of IOP on the cell viability of OA-induced HepG2 cells. Each value represents the mean of six replicates, and error bars indicate standard deviation (±SD). Different letters show the significant difference between different groups of cells (*p* < 0.05).

The influence of OA and IOP on cell viability were detected by MTT assay. With the concentration of OA increased, the cell viability decreased. When the OA concentrations were lower than 0.2 mM, the cell viability had no significant effect during 24 h cultivation, and the cell viability of HepG2 reached 87.12%. In the following experiments, 0.2 mM OA concentration was chosen to induce a lipid accumulation cell model (Figure 2a). Then, different contents of IOP were added into the cell culture medium containing 0.2 mM OA. Figure 2b showed that after incubation for 24 h, IOP treatment at 20, 40, and 60 mg/L did not damage HepG2 cells. Higher concentrations of IOP (80 mg/L) reduced cell viability (*p* < 0.05). In the following experiments, 0.2 mM OA concentration was selected to establish cell models. The selected low, medium, and high concentrations of IOP were 20, 40, and 60 mg/L, respectively.

### 3.4. Oil Red O Staining in OA-Induced HepG2 Cells

**Figure 3:**
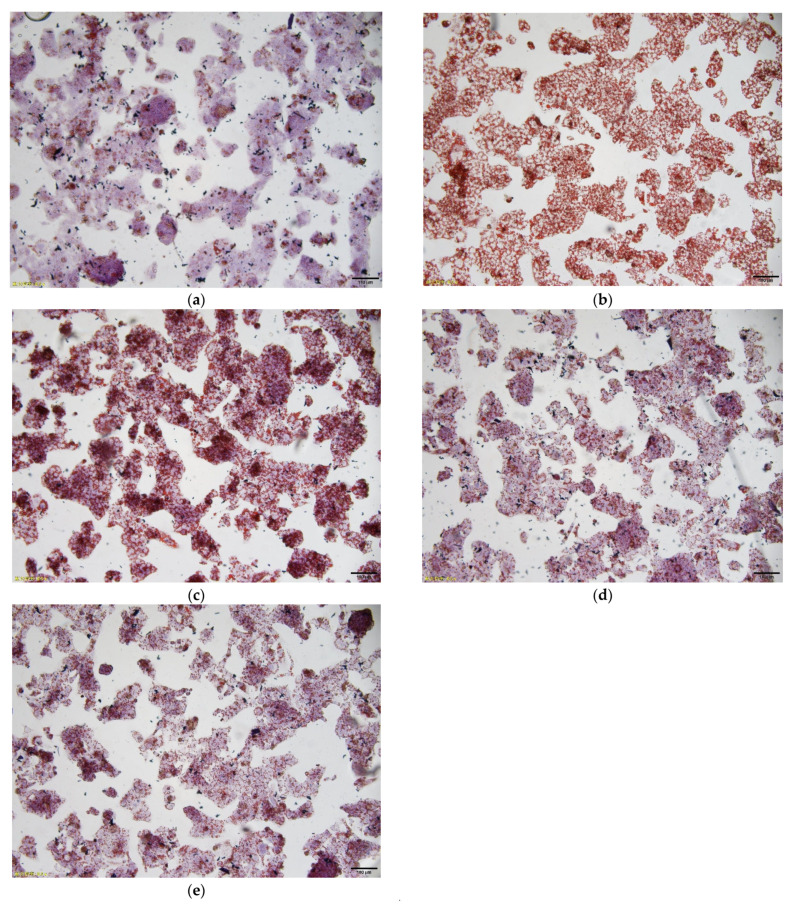
Oil red O staining in OA-induced HepG2 cells. (**a**) NC group (HepG2 cells); (**b**) OA group (0.2 mM OA-induced HepG2 cells); (**c**) LIOP group (0.2 mM OA + 20 mg/L IOP); (**d**) MIOP group (0.2 mM OA + 40 mg/L IOP); and (**e**) HIOP group (0.2 mM OA + 60 mg/L IOP). Bars × 400.

Figure 3 showed different levels of red lipid droplets in the OA-induced HepG2 cells under different IOP concentrations. The NC group was cultured in normal DMEM medium, the cell status was normal, and red lipid droplets were almost invisible. The OA group was cultured in DMEM medium containing 0.2 mM OA, the lipid accumulation was serious, and large red lipid droplets were noted (Figure 3b). IOP can reduce excessive lipids in HepG2 cells caused by OA. Compared with the OA group, lipid accumulation was significantly reduced in IOP-treated groups. When the IOP concentration increased in HepG2 cells, the red lipid droplets became smaller and lighter gradually. Compared with the OA group, HepG2 cells treated with 60 mg/L IOP exhibited significantly decreased lipid droplets, suggesting that IOP prevented HepG2 cells from excessive lipid accumulation in vitro (Figure 3e).

### 3.5. Effect of IOP on TG, TC, HDL-C, and LDL-C Contents of OA-Induced HepG2 Cells

**Figure 4:**
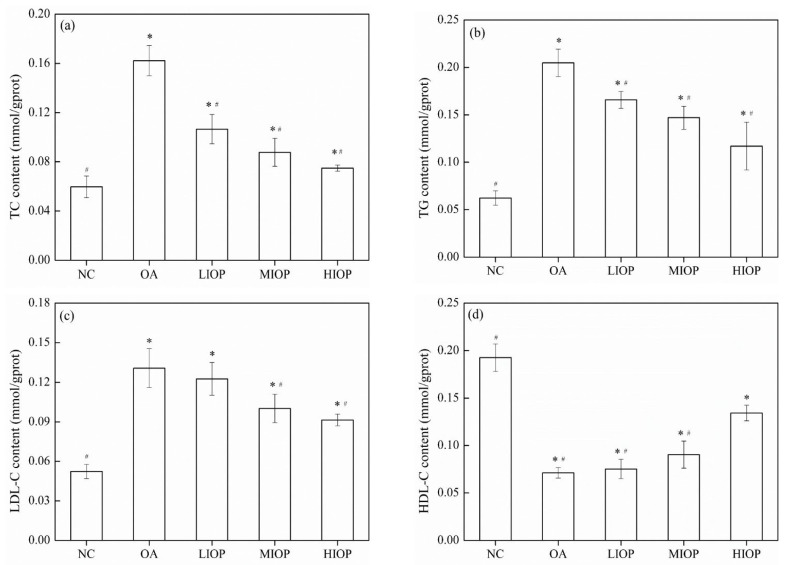
Effect of IOP on TG, TC, HDL-c, and LDL-c contents of OA-induced HepG2 cells. (**a**): TG; (**b**): TC; (**c**): HDL-C; (**d**): LDL-C. Each value represents the mean of six replicates, and error bars indicate standard deviation (±SD). * *p* < 0.05, versus the NC group; # *p* < 0.05, versus the OA group.

The effects of IOP on lipid metabolism in OA-induced HepG2 cells were tested at the following groups: LIOP, MIOP, and HIOP (Figure 4). Compared with the OA group, all the IOP groups decreased the TC and TG contents significantly (*p* < 0.05). TC and TG contents in the HIOP group were 0.075 ± 0.002 and 0.117 ± 0.025 mmol/g prot lower, respectively, than those of the OA group (0.162 ± 0.012 and 0.205 ± 0.014 mmol/g prot, respectively). When 60 mg/L of IOP was added to the cell culture medium, the content of LDL-C was significantly reduced and the content of HDL-C significantly increased (*p* < 0.05) in HepG2 cells (Figure 4). The amount of TC, TG, and LDL-C decreased following 60 mg/L of IOP treatment compared with those of the OA-induced HepG2 cells.

### 3.6. Effect of IOP on Body Weight of Mice

**Figure 5:**
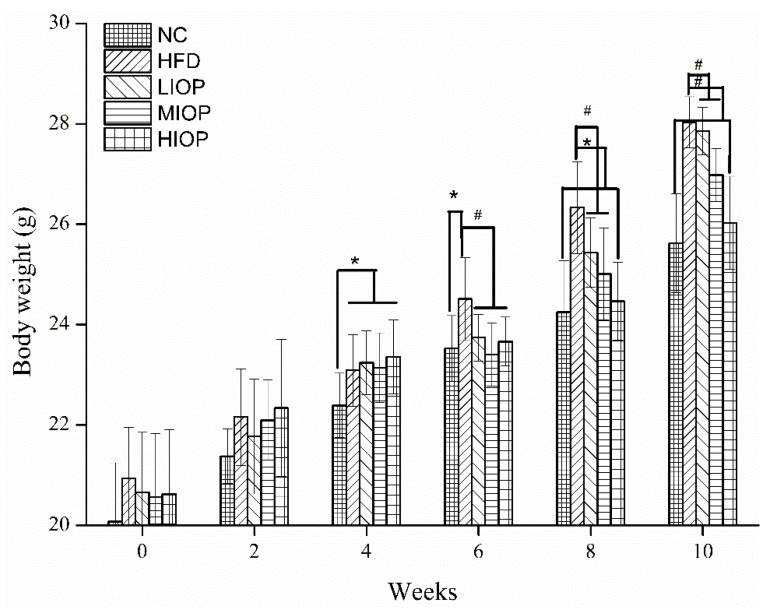
Effects of IOP on the body weight of mice. The data are represented as mean ± SD (*n* = 8). * *p* < 0.05, versus the NC group; # *p* < 0.05, versus the HFD group.

As shown in Figure 5, the body weight of the HFD group increased faster than that of the NC group, indicating that feeding a high-fat diet can significantly increase body weight. After 6 weeks of IOP intervention, the HIOP group was significantly different from the HFD group (*p* < 0.05). After 10 weeks of IOP intervention, the body weight of mice in the LIOP, MIOP, and HIOP groups was significantly lower than the HFD group (*p* < 0.05). It can be seen that IOP can effectively reduce the body weight gain of mice induced by a high-fat diet.

### 3.7. Effect of IOP on the Biochemical Indicators of Mice

As shown in Table 3, the serum TC, TG, and LDL-C concentrations of mice in the HFD group were significantly higher than those in the NC group (*p* < 0.05). Compared with the HFD group, the serum TC, TG, and LDL-C concentrations of LIOP, MIOP, and HIOP mice decreased significantly (*p* < 0.05). This showed that IOP had an obvious lipid-lowering effect. In particular, compared with the HFD group, the TC concentration of HIOP group decreased by 40.0%, the TG concentration decreased by 24.8%, and the LDL-C concentration decreased by 30.1%. The serum HDL-C concentration of mice in the HFD group was significantly lower than that in the NC group (*p* < 0.05). Compared with the HFD group, the serum HDL-C concentration of rats in the LIOP, MIOP, and HIOP groups increased significantly by 22.2%, 30.3%, and 49.2%, respectively (*p* < 0.05). This shows that IOP can increase the level of serum HDL-C, and is not affected by the dose of IOP.

### 3.8. Liver Histological Images

**Figure 6:**
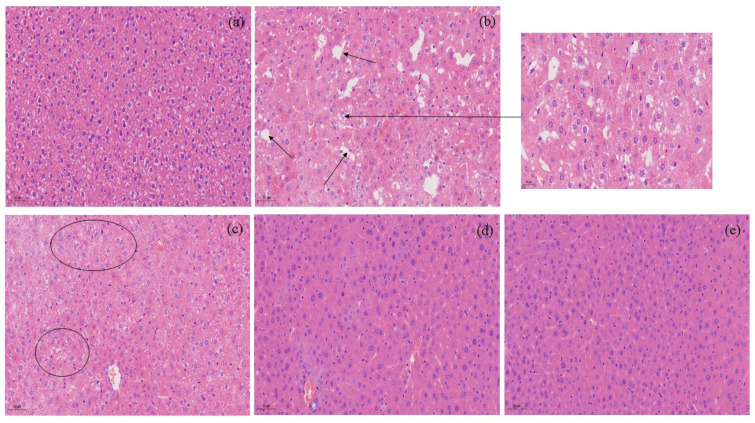
Liver histological images of mice. (**a**) NC group; (**b**) HFD group; (**c**) LIOP group; (**d**) MIOP group; (**e**) HIOP group. The lesion area is marked with arrows and enlarged.

In the NC group, the hepatocytes were arranged neatly, the structure of liver lobules was clear, the cells had no steatosis, and there was no inflammatory cell infiltration in the portal area. In the HFD group, hepatocytes were swollen, there were lipid droplet vacuoles of different sizes in the cells, the nucleus was marginalized, and there was inflammatory cell infiltration in the portal area. Compared with the HFD group, the MIOP group and HIOP group have significantly reduced steatosis and reduced inflammatory cells (Figure 6).

### 3.9. Effect of IOP on the mRNA Expression of Mice

**Figure 7:**
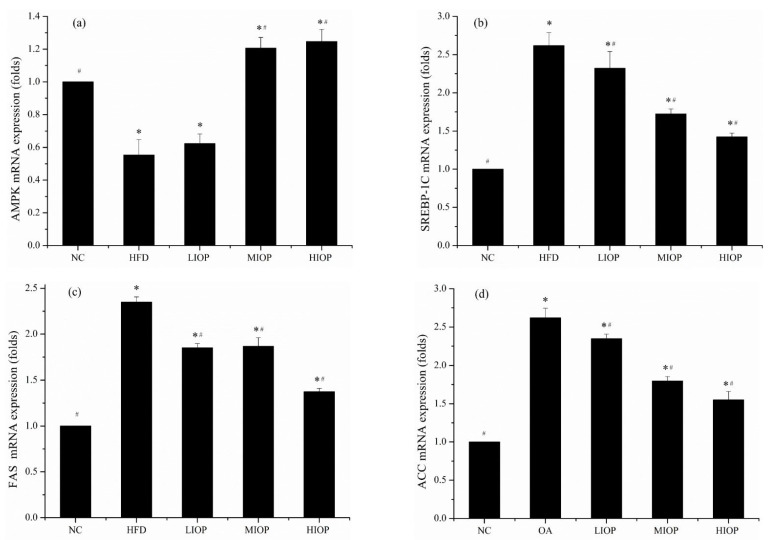
Effect of IOP on mRNA expression of mice livers. (**a**) AMPK; (**b**) SREBP-1C, (**c**) FAS; (**d**) ACC. Each value represents the mean of eight mice, and error bars indicate the standard deviation (±SD). * *p* < 0.05, versus the NC group; # *p* < 0.05, versus the HFD group.

The expression of lipid synthesis-related genes is shown in Figure 7a–d. The mRNA expression of AMPK was downregulated in the HFD group (*p* < 0.05), while HIOP reversed the downregulated expression (*p* < 0.05). High-fat diet upregulated the expression of genes related to lipid synthesis. For example, the expression of SREBP-1C increased in the HFD group. The change trend of ACC and FAS were the same as SREBP-1C. Different concentrations of IOP had different effects on the mRNA expression of mice. Compared with the HFD group, the HIOP group showed a significantly decreased expression of SREBP-1C. At the same time, the mRNA expression of ACC and FAS also decreased (*p* < 0.05).

### 3.10. Effect of IOP on Protein Expression of Mice

**Figure 8:**
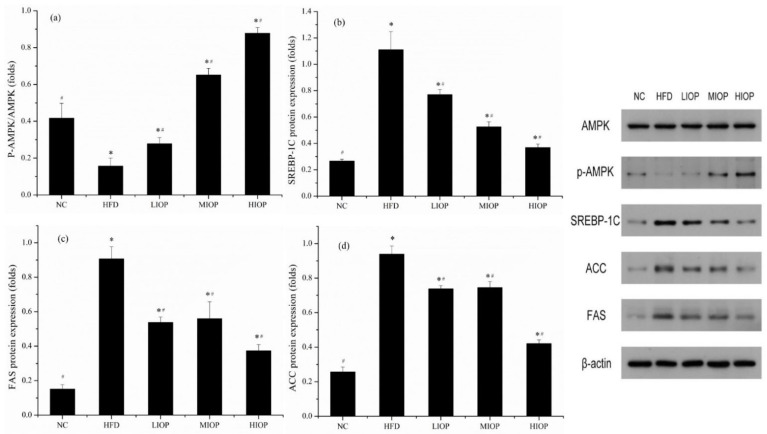
Effect of IOP on protein expression of mice livers. (**a**) P-AMPK/AMPK; (**b**) SREBP-1C, (**c**) FAS; (**d**) ACC. Each value represents the mean of eight mice, and error bars indicate the standard deviation (±SD). * *p* < 0.05, versus the NC group; # *p* < 0.05, versus the HFD group.

Protein expression of AMPK, SREBP-1C, FAS, and ACC is shown in Figure 8a–d. IOP affected the content of lipids and the content of liposynthesis enzymes in mice. The AMPK and p-AMPK protein expressions were investigated. Compared with the HFD group, the protein expression level of p-AMPK in the LIOP, MIOP, and HIOP groups was significantly increased (*p* < 0.05). Compared with the NC group, the protein expression levels of SREBP-1C, FAS, and ACC in the HFD group were significantly increased (*p* < 0.05). Compared with the HFD group, LIOP, MIOP, and HIOP can significantly reduce SREBP-1C, FAS and ACC protein expression level of mice liver (*p* < 0.05). At the same time, the mRNA expression levels of SREBP-1C, FAS and ACC were consistent with their protein expression levels. IOP can promote AMPK and inhibit SREBP-1C, FAS, and ACC protein synthesis.

## 4. Discussion

The biological activities of polysaccharides have become research hotspots [27]. Plant polysaccharides have biological activities, such as immune regulation, antiviral, hypoglycemic, hypolipidemic, and antioxidation effects, which participate in various physiological metabolisms of the human body [28]. IOP was an acidic heteropolysaccharide with a rough surface. According to Huang’s study, IOP3a was mainly composed of rhamnose, mannose, glucose, and galactose with a molar ratio of 0.3:4.6:2.3:1.0. IOP3a was a polysaccharide with Mw of 44kDa and strong antioxidant activity [29]. Du found that IOP60 extracted from *Inonotus obliquus* sclerotia included 1-fucose, 1-rhamnose, 1-arabinose, d-galactose, d- glucose, d-xylose, d-mannose, d-fructose, d-ribose, d-galacturonic acid, and d-glucuronic acid [30]. Similar to the previous studies, IOP was an acidic polysaccharide, which included glucose, rhamnose, and ribose. Mannose, fructose, and other monosaccharides were not detected, which may be caused by differences in the extraction method and raw materials. Wang’s study showed that I. obliquus polysaccharides-chromium (III) complex (UIOPC) with Mw of 115 kDa had anti-diabetic effects. According to the FTIR spectra analysis, UIOPC contains C-H, O-H, and C-O, which was consistent with our study. However, -CH3 and Cr-OH were not detected [31]. According to SEM images, native sample IOPS was irregular with different sizes. However, after modification, the surface of Ac-IOPS was rough. These differences may be caused by different chemical modifications [32]. The structure of polysaccharide affected its functional properties. A loose and disordered structure of IOP might remarkably reduce the diffusion rate of cholesterol. IOP had potential lipid-lowering effects due to its special structure and may be used as nutritional supplements to enhance physical fitness and assist in the treatment of metabolic diseases. For example, *Agaricus blazei Morrill* acidic polysaccharide had hypolipidemic effects in vitro [33]. Similar to our research results, IOP may be acidic polysaccharides, which had good functional properties.

OA is an unsaturated fatty acid that can cause lipid accumulation in the liver of patients with non-alcoholic fatty liver disease. The high-fat HepG2 cell model induced by oleic acid is a suitable simple conventional steatosis in vitro model, showing the pathological characteristics of lipid accumulation [34]. In this study, a cell model of fatty degeneration induced by 0.2 mM OA was successfully established. However, high polysaccharide content was not suitable for cell growth. Our research also found similar results as before, in that high content of *Cyclocarya paliurus* polysaccharides accelerated RAW 267.7 cell apoptosis [35]. Therefore, according to the MTT experiment of cell survival rate, 80 mg/L IOP was not suitable for cell growth. IOP concentrations of 20, 40, and 60 mg/L were chosen for the experiment. Oil Red O can specifically bind to triglycerides in cells and stain the fat in the cells red [36]. After OA was absorbed by the cells, TG content increased and lipid droplets accumulate in the cells. The experimental results of oil red staining indicated that *I. obliquus* polysaccharide can reduce the accumulation of fat in hepatocytes induced by OA in vitro and mainly affect lipid synthesis and inflammation, indicating that IOP had hypolipidemic activity. Sufficient evidence showed that biologically active molecules had lipid-lowering effects. For instance, genistein dose-dependently decreased the TG content in cells [37]. Our results were similar to those of Li, where polysaccharides from *Agaricus blazei* had similar lipid-lowering effects by regulating fat synthesis related protein expression, thereby reducing TC and TG contents in HepG2 cells [38]. In vitro experiments have proved that IOP had lipid-lowering activity. Therefore, a high-fat diet animal model was established to study the molecular mechanism of IOP’s lipid-lowering effect.

The prevalence of obesity, hyperlipidemia, and non-alcoholic fatty liver disease is increasing worldwide. The long-term excessive intake of high-fat foods will interfere with lipid metabolism [39]. The most intuitive indicator is the content of TC and TG in the liver [40]. TC, TG, and LDL-C increase and HDL-C decrease may lead to fat accumulation and increase the risk of non-alcoholic fatty liver disease [41]. In our study, IOP decreased the bodyweight and blood lipid levels in high-fat diet mice.

**Figure 9:**
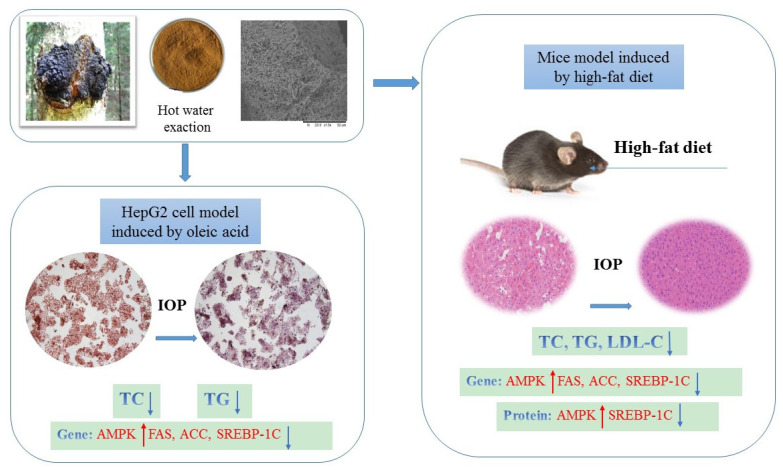
Mechanisms of IOP-mediated lipid-lowering effects.

In vivo experiments proved that IOP may regulate the AMPK/SREBP-1C/FAS/ACC signaling pathway. AMPK is the key to the regulation of bioenergy metabolism and the core of the research on some metabolic-related diseases [42]. Thus, as the concentration of IOP increased, the AMPK mRNA expression of mice livers increased. This result was similar to that of Jin that *Schisandra* polysaccharides regulated the expression of AMPK in a dose-dependent manner [43]. IOP may reduce the blood lipid by activating AMPK and promote the oxidation of fatty acids. SREBP-1C plays an important role in liver lipid metabolism, and it is involved in the transcription of almost all liver triglyceride and fatty acid synthesis genes. Importantly, SREBP-1C can participate in the regulation of the expression of ACC, FAS and other genes related to fat synthesis. ACC is considered to be a rate-limiting enzyme in the fatty acid synthesis pathway [44]. ACC is a key regulator of fatty acid biosynthesis and could catalyze the conversion of acetyl coenzyme A into malonyl coenzyme A [45]. FAS is a kind of key lipogenic enzyme. Malonyl-CoA is closely related to obesity [46]. ACC and FAS usually cooperate with each other and work together. In this study, the results showed that after the mice fed by high-fat diet, mRNA levels of SREBP-1C, ACC, and FAS increased significantly, which was consistent with the result of increased lipid deposition in the mice liver. After HIOP intervention, compared with the HFD group, the mRNA levels of SREBP-1, FAS, and ACC all decreased significantly, indicating that IOP can reduce bodyweight and blood lipid level by inhibiting fat synthesis. A lot of evidence shows that plant polysaccharides can improve lipid metabolism by affecting SREBP-1C expression and finally inhibiting ACC and FAS expression. For example, pectin from steamed ginseng decreased TC and TG levels increased by inhibiting ACC expression [47]. In our current research, increased protein concentration of p-AMPK and the decreased protein concentration of SREBP-1C caused by IOP suggested the mitigation of lipid accumulation in high-fat diet mice. Biologically active molecules can activate the AMPK pathway to achieve lipid-lowering effects [48]. AMPK downregulates the activity of fatty acid synthesis-related enzymes and prevents lipid production by inhibiting the expression of lipid production-related factor SREBP-1C; as such, it plays a therapeutic role in diseases of abnormal liver lipid metabolism [49]. The major strength of this work is our finding that IOP could effectively reduce the TC and TG contents and downregulate SREBP-1C, ACC, and FAS gene expression. We speculated that IOP exerted a potential lipid-lowering effect by regulating AMPK protein expression and SREBP-1C protein expression (Figure 9).

## 5. Conclusions

IOP was a heteropolysaccharide with a molecular weight of 42.28 kDa. IOP had a strong effect on decreasing the TG, TC, and LDL-C levels and increasing the HDL-C levels in OA-induced HepG2 cells. In the oil red-stained samples, the red fat droplets were significantly reduced. Moreover, IOP reduced the bodyweight and blood lipid levels of mice induced by high-fat diet. A high dose of IOP significantly reduced steatosis and inflammatory cells. IOP stimulated the gene expression and protein expression of AMPK, SREBP-1C, FAS, and ACC in mice livers. In this study, cell experiments and mice experiments verified that IOP has lipid-lowering effects both in vivo and in vitro, suggesting that IOP as a functional food may have a certain potential for development in lipid lowering.

## Figures and Tables

**Table 1:** Treatment for experimental mice.

Group	Diet	Treatment
NC (Normal control)	Normal diet	0.2 mL PBS per day
HFD (High-fat diet)	High-fat diet	0.2 mL PBS per day
LIOP (Low dose of IOP)	High-fat diet	0.2 mL PBS + 200 mg/kg.bw IOP per day
MIOP (Medium dose of IOP)	High-fat diet	0.2 mL PBS + 400 mg/kg.bw IOP per day
HIOP (High dose of IOP)	High-fat diet	0.2 mL PBS + 600 mg/kg.bw IOP per day

Notes: normal diet (energy: 4.0 kcal g^−1^, 20% kcal from protein, 12% kcal from fat, 68% kcal from carbohydrates) and high-fat diet (energy: 4.7 kcal g^−1^, 16% kcal from protein, 42% kcal from fat, 43% kcal from carbohydrates).

**Table 2:** Primer sequences used for RT-PCR.

Genes	Primer Sequences	
	Forward (5′–3′)	Reverse (5′–3′)
GAPDH	CTCTCTGCTCCTCCTGTTCG	ACGACCAAATCCGTTGACTC
FAS	GTACACAGACAAAGCCCATTTT	TTTGGTTTACATCTGCACTTGG
AMPK	CAACTATCGATCTTGCCAAAGG	AACAGGAGAAGAGTCAAGTGAG
ACC	TACCTTCTTCTACTGGCGGCTGAG	GCCTTCACTGTTCCTTCCACTTCC
SREBP-1C	CTGTGTGACCTGCTTCTTGT	CTCATGTAGGAACACCCTCC

**Table 3:** Effect of IOP on the levels of serum lipids of experimental mice.

Group	TC (mmol/L)	TG (mmol/L)	LDL-C (mmol/L)	HDL-C (mmol/L)
NC	2.57 ± 0.17 ^d^	2.27 ± 0.30 ^b^	0.93 ± 0.16 ^c^	2.86 ± 0.40 ^a^
HFD	6.15 ± 0.54 ^a^	3.15 ± 0.36 ^a^	1.63 ± 0.23 ^a^	1.85 ± 0.14 ^d^
LIOP	5.08 ± 0.44 ^b^	2.35 ± 0.19 ^b^	1.36 ± 0.27 ^b^	2.26 ± 0.44 ^c^
MIOP	4.85 ± 0.27 ^b^	2.30 ± 0.34 ^b^	1.19 ± 0.34 ^bc^	2.41 ± 0.38 ^bc^
HIOP	3.69 ± 0.60 ^c^	2.37 ± 0.20 ^b^	1.14 ± 0.15 ^bc^	2.76 ± 0.37 ^ab^

Different letters show the significant difference between different groups of mice body weight. Data are expressed as mean ± standard deviation (*n* = 8). Different letters show the significant difference between different groups of mice (*p* < 0.05).

## Data Availability

Data in the project are still being collected, but all data used in the study are available by contacting the authors.

## Supplementary Materials

The following are available online at https://www.mdpi.com/article/10.3390/foods10123085/s1, Figure S1. Flow chart of the preparation of IOP, Figure S2. Response surface plots showing the effects of variables on the extraction yield of IOP. Table S1. Factors and levels used in a Box–Behnken design, Table S2. Box–Behnken experimental design and the results for the yield of IOP, Table S3. ANOVA for the response surface quadratic model.

## Abbreviations

ACC	Acetyl-coenzyme (Co) A carboxylase
AMPK	Adenosine 5′-monophosphate (AMP)-activated protein kinase
DMEM	Dulbecco’s Modified Eagle’s Medium
DMSO	Dimethyl sulfoxide
ELISA	Enzyme-linked immunosorbent assay
FAS	Fatty acid synthase
FBS	Fetal bovine serum
GPC	Gel permeation chromatography
HDL-C	High density lipid-cholesterol
LDL-C	Low density lipid-cholesterol
MTT	3-(4,-Dimethyl-2-thiazolyl)-2,-diphenyl-2-H-tetrazolium bromide
OA	Oleic acid
PBS	Phosphate-buffered saline
SREBP-1C	Sterol regulatory element binding protein-1C
TC	Total cholesterol
TG	Triglyceride
