# Supplementary material for: Lipid-Lowering Effects of Inonotus obliquus Polysaccharide In Vivo and In Vitro
Source: Foods. 2021 Dec 12;10(12):3085. doi: 10.3390/foods10123085 (PMC8700920; doi:10.3390/foods10123085)
Supplement: Supplementary file 1 [file foods-10-03085-s001.zip › foods-1482264-supplementary.pdf]

Supplementary files

Response surface analysis

The optimized parameters were extraction time 3.06 h, extraction temperature 80.16 °C and water material ratio 30.81: 1. Under the conditions, the yield of IOP was 5.37 ± 0.69%.

Tables

Table S1. Factors and levels used in Box-Behnken design.

Factors	Levels		
	-1	0	1
A:Temperature (°C)	70	80	90
B:Time (h)	2	3	4
C:Water material ratio	20	30	40

Table S2. Box-Behnken experimental design and the results for the yield of IOP

Run	A:Temperature (°C)	B:Time (h)	C:Water materiral ratio(ml/g)	Yield (%)
1	70	2	30	2.55
2	90	2	30	2.75
3	70	4	30	2.76
4	90	4	30	3.07
5	70	3	20	3.16
6	90	3	20	2.68
7	70	3	40	3.09
8	90	3	40	3.33
9	80	2	20	3.43
10	80	4	20	3.29
11	80	2	40	3.28
12	80	4	40	3.81
13	80	3	30	5.47
14	80	3	30	5.63
15	80	3	30	5.12
16	80	3	30	5.20
17	80	3	30	5.41

Table S3. ANOVA for response surface quadratic model

Sourse	Sum of squares	df	Mean square	F value	p-value Prob>F
Model	19.5	9	2.17	60.96	<0.0001**
A-temperature	9.113E-003	1	9.113E-003	0.26	0.6282
B-time	0.11	1	0.11	2.98	0.1281
C-water material ratio	0.11	1	0.11	3.17	0.1180
AB	3.025E-003	1	3.025E-003	0.085	0.7789
AC	0.13	1	0.13	3.65	0.0978
BC	0.11	1	0.11	3.16	0.1188
A2	9.29	1	9.29	261.42	<0.0001**
B2	5.08	1	5.08	142.82	<0.0001**
C2	2.80	1	2.80	78.78	<0.0001**
Residual	0.25	7	0.036		
Lack of fit	0.078	3	0.026	0.61	0.6421
Pure error	0.17	4	0.043		
Cor Total	19.75	16			
R-Squared=0.9874 Adj R-Squared=0.9712 Pred R-Squared=0.9231 Adeq Precision=18.706					

Figures

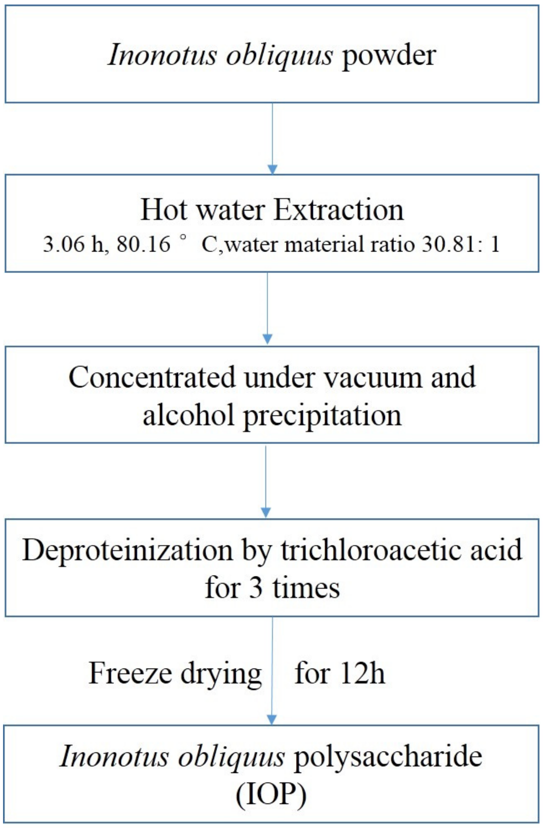

Figure S1. Flow chart of preparation of IOP

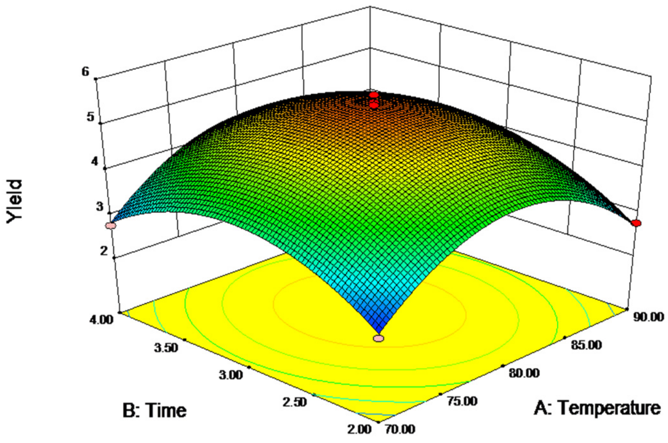

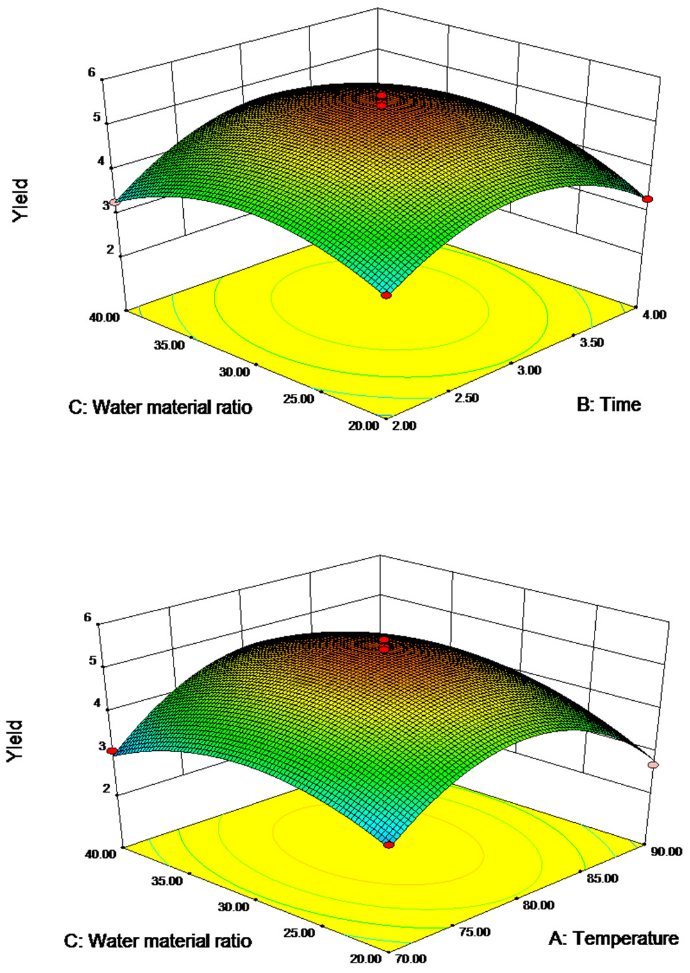

Figure S2. Response surface plots showing effects of variables on the extraction yield of IOP. A: Temperature (°C), B: Time (h) and C: Water material ratio (mL/g).
